# Preparing raw clinical data for publication: guidance for journal editors, authors, and peer reviewers

**DOI:** 10.1186/1745-6215-11-9

**Published:** 2010-01-29

**Authors:** Iain Hrynaszkiewicz, Melissa L Norton, Andrew J Vickers, Douglas G Altman

**Affiliations:** 1 BioMed Central, 236 Gray's Inn Road, London WC1X 8HL, UK; 2 Department of Epidemiology and Biostatistics, Memorial Sloan Kettering Cancer Center, 1275 York Avenue, NY, NY 10021, USA; 3 Centre for Statistics in Medicine, University of Oxford, Wolfson College Annexe, Oxford OX2 6UD, UK

## Abstract

In recognition of the benefits of transparent reporting, many peer-reviewed journals require that their authors be prepared to share their raw, unprocessed data with other scientists and/or state the availability of raw data in published articles. But little information on how data should be prepared for publication - or sharing - has emerged. In clinical research patient privacy and consent for use of personal health information are key considerations, but agreed-upon definitions of what constitutes anonymised patient information do not appear to have been established. We aim to address this issue by providing practical guidance for those involved in the publication process, by proposing a minimum standard for de-identifying datasets for the purposes of publication in a peer-reviewed biomedical journal, or sharing with other researchers. Basic advice on file preparation is provided along with procedural guidance on prospective and retrospective publication of raw data, with an emphasis on randomised controlled trials.

In order to encourage its wide dissemination this article is freely accessible on the BMJ and Trials journal web sites.

## Background

Many peer-reviewed journals' instructions for authors require that authors should be prepared to share their raw (that is, unprocessed) data with other scientists on request. Although data sharing is commonplace in some scientific disciplines and is a requirement of a number of major research funding agencies' policies, this culture has not yet been widely adopted by the clinical research community. Some journals have appealed to their authors to increase the availability of medical research data [1-3], recognising the benefits of such transparency. These benefits are well documented and include replication of previous findings, comparisons with independent datasets, testing of additional hypotheses, teaching, and patient safety [3-6]. Moreover, patients themselves are increasingly seeing the benefits of openly sharing their experiences with others [7].

Online journals with unlimited space now provide the platform for publishing large, raw datasets as supplementary material [5,8], but a common concern is confidentiality. If there is any doubt over anonymity, publishing data that have arisen from the doctor-patient or researcher-participant relationship will raise issues of privacy unless explicit and properly informed consent to all of the intended uses of that data has been obtained. The International Committee of Medical Journal Editors' *Uniform Requirements for Manuscripts Submitted to Biomedical Journals *require that patient privacy be protected, and maintaining confidentiality and privacy is ingrained in various legal statutes such as the UK Data Protection Act and the Health Insurance Portability and Accountability Act (HIPAA) in the US [9].

In Europe, the Data Protection Directive (Directive 95/46/EC) provides some harmony in data protection legislation, but in the US there is no overarching data protection law. Therefore, in an increasingly global research and publishing industry, universally agreed definitions as to what constitutes anonymised patient information would benefit clinical researchers. The HIPAA provides a list of 18 items that need to be removed from patient information in order for it to be considered anonymous for the purposes of sharing information between the "covered entities" specified in the act, but the list was not designed with publication in biomedical journals in mind. A number of publications from UK bodies provide some form of guidance on identifying information [10-13], but none is as explicit as the HIPAA.

This article aims to provide practical guidance for those involved in the publication process by proposing a minimum standard for anonymising (or de-identifying) data for the purposes of publication in a peer-reviewed biomedical journal or sharing with other researchers, either directly, where appropriate, or via a third party. Basic advice on file preparation is also provided, along with procedural guidance on prospective and retrospective publication of raw clinical data. Although the focus of this discussion is on data from randomised trials, the same issues of confidentiality apply to data from any research study involving human subjects, including cohort, case-control, and case series designs.

### Data preparation guidance

#### What is the dataset?

For the purposes of this guidance, the dataset is the aggregated collection of patient observations (including sociodemographic and clinical information) used for the purposes of producing the summary statistical findings presented in the main report of the research project, whether previously published or not.

Data are almost always collected at a greater level of detail than are reported in a journal article. For example, each participant in a pain study may complete a pain diary twice a day for 30 days, with the authors reporting "mean post-treatment pain" for one or more groups of participants. Similarly, a quality of life questionnaire may include a large number of questions divided into domains such as physical, mental, emotional, and social wellbeing.

Here we define a dataset as that containing the minimum level of detail necessary to reproduce all numbers reported in the paper. The dataset for the pain trial, for example, might therefore consist of one value per individual for mean post-treatment pain rather than 30 values for pain levels on each day. However, if more detailed, underlying data are available and can be shared then that should also be encouraged, provided the data conform to the same standards--as proposed in this article--as the main dataset. If possible, authors should present all outcomes and variables, regardless of significance.

#### Anonymisation

A list of 28 patient identifiers has been formulated, based on information aggregated from policy documents and research guidance from major UK and US funding agencies, governmental health departments and statutes, and three internationally recognised publication ethics resources for editors of biomedical journals [9-16]. This list is provided in Table 1.

**Table 1:** Aggregated list of potential patient identifiers in datasets

Identifier (information sources)	Comments
**Direct**	
Name [9-16]	
Initials [14]	
Address, including full or partial postal code [9-16]	
Telephone or fax numbers or contact information [9,11,13,16]	
Electronic mail addresses [9]	
Unique identifying numbers [9-16]	Generalised HIPPA item 7-10, 18
Vehicle identifiers [9]	
Medical device identifiers [9]	
Web or internet protocol addresses [9]	
Biometric data [9]	
Facial photograph or comparable image [9,11,12,14]	
Audiotapes [12]	
Names of relatives [11]	
Dates related to an individual (including date of birth) [9,10,12,16]	
**Indirect--may present a risk if present in combination with others in the list**	
Place of treatment or health professional responsible for care [11,16]	Could be inferred from investigator affiliations
Sex [10]	
Rare disease or treatment [11]	
Sensitive data, such as illicit drug use or "risky behaviour" [16]	
Place of birth [11,16]	
Socioeconomic data, such as occupation or place of work, income, or education [10,11,13,16]	MRC requirement is for "rare" occupations only
Household and family composition [16]	
Anthropometry measures [16]	
Multiple pregnancies [16]	
Ethnicity [10]	
Small denominators--population size of <100 [15]	
Very small numerators--event counts of <3 [15]	
Year of birth or age (this article)	Age is potentially identifying if the recruitment period is short and is fully described
Verbatim responses or transcripts [16]	

Types of identifying information have been classified as either direct or indirect. Publication of any direct identifiers places individuals in the dataset at risk of being identified. Although none of the indirect identifiers on its own would point to an individual, a dataset with several indirect identifiers, especially those relating to attributes, might do. The consensus of the authors, and working group members acknowledged in the current manuscript, is that a dataset including three or more indirect identifiers should be assessed by an independent researcher or ethics committee to evaluate the risk that individuals might be identifiable. If the risk of identification is considered non-negligible, before publication can proceed approval should be sought from a relevant advisory body (see below). An explicit justification for publication of a dataset with three or more indirect identifiers should be given by the researcher--as an annotation to the dataset and in any accompanying articles. This should include the name of any oversight bodies consulted.

#### Use of dates relevant to individuals

In circumstances where it is essential for the scientific validity of the study to include dates, such as dates of treatment (a direct identifier), data must be presented in such a way that is unlikely to affect statistical analyses but preserves anonymity. For example, one could add or subtract a small, randomly chosen number of days to all dates, so that the true dates are not published. In cases where it is necessary to include dates, this fact and any supporting information should be disclosed on submission to the journal.

#### File preparation

Authors should provide a clean, well annotated dataset in a suitable format so that statistical analyses could be conducted. By "clean," we mean reviewed systematically for duplicates, errors, and missing data; by "well annotated," we mean that sufficient information is given about each variable to allow replication of the originally published results. For example, the dataset included as supplementary material by Vickers [5] includes a brief description of the study and data and a detailed explanation of each variable on the dataset. It is recommended that file formats be as general as possible. Microsoft Excel is widely used and delimited text format is universally convertible, so these formats are preferable to files saved in formats specific to statistical software such as SAS or STATA. If a dataset may be updated in the future--for example, in cancer studies where follow-up is continued over many years--it could be given a version number or date.

#### Copyright

Where datasets are being published as supplementary material in a journal that requires transfer of copyright to the publisher, it is recommended the supporting data be separated from the article itself and that transfer of copyright for the data is not required as a condition of publication. Of note, there is no protection by intellectual property law on data that are gathered for research purposes [17]--facts themselves are not copyrightable, only the way in which they are expressed.

#### Prospective data publication

With the increasing prevalence of data sharing policies from research funding agencies, researchers should be encouraged to make allowances for data sharing or publication when preparing study protocols. Although consent is not required in law to process anonymised data, ideally informed consent should be obtained from research participants for the publication of suitably anonymised raw data, as part of the recruitment process, for all new studies. Researchers should inform participants of all possibilities for the use of their information and allow them to choose. Participants who do not agree to publication of potentially identifying information may need to be removed from the dataset [16]. Approval or consent should include use of the data in subsequent meta-analyses. Research ethics committees should also encourage researchers to include details of the intention to publish data in the study information sheets that are provided to study participants, and to ensure that safeguards are in place to protect patient privacy. In the absence of mandates for data sharing or publication, research funding agencies should give greater scrutiny to data sharing plans referred to in their policy documents and check their enforcement.

#### Retrospective data publication

There will be instances when researchers wish to publish a dataset retrospectively. This might be from current clinical research that was conducted without explicit consent for data sharing or publication from the participants (due to a lack of specific requirements of funders or regulators) or use of data from a historic piece of research conducted before data sharing policies were established.

In such cases, researchers may publish raw data if it is clear and demonstrable that there is no threat to anonymity--for example, if the dataset includes no direct identifiers and fewer than three indirect identifiers. If it is not certain that data are completely anonymous, and where consent of all participants is not possible, a careful case-by-case assessment must be made--taking into account public interest and scientific imperative for publication--before publishing the data. Where there is a risk of identification, we recommend authors consult local ethics committees about their wish to publish their raw data in a freely accessible manner before submitting it for publication. When the relevant committee no longer exists, the authors are encouraged to consult an appropriate national advisory body. In the UK, for example, the National Information Governance Board for Health and Social Care (NIGB), formerly the Patient Information Advisory Group, provides advice on issues of national significance involving the use of patient information. The NIGB includes the Ethics and Confidentiality Committee. Such bodies may not be in a position to approve the decision to publish raw data, but they could provide a valuable opinion. Advice may also be sought from the Caldicott Guardian (a person responsible for protecting patient confidentiality) or equivalent person within the author's institution. In the US, a research ethics consultation could be considered in addition to institutional review board approval.

A case-by-case judgment, whether this is by an advisory body or the journal editor, will need to take into account the sample size, the ways in which results will be published and used, and all other circumstances of the study. For example, the fact that research findings are increasingly being published in open access journals, so that a published dataset would be visible to anyone with an internet connection, arguably makes any issues of confidentiality and anonymity even more important.

#### Preparing for journal submission--statement in submitted manuscript

Authors should be asked to state in their manuscript if informed consent for data publication has been obtained. If consent was not obtained, authors should be asked to state the reasons for this and the name of the body that gave approval or any guidance adhered to in preparing their data for publication. In practice, authors could make one of three statements:

1 Consent for publication of raw data obtained from study participants

2 Consent for publication of raw data not obtained but dataset is fully anonymous in a manner that can easily be verified by any user of the dataset. Publication of the dataset clearly and obviously presents minimal risk to confidentiality of study participants

3 Consent for publication of raw data not obtained and dataset could in theory pose a threat to confidentiality.

For statements 2 and 3, authors should also provide:

• Reasons why it was not possible to obtain consent

• Reasons why publication of data constituted a negligible risk to confidentiality *or *reasons why benefit of publishing data outweighs a non-negligible risk to confidentiality, *plus *the name of an oversight body consulted for approval of publication or guidance.

### Alternatives to journal publication

There will be circumstances where raw data cannot be published in journals, because of policy or space restrictions, but alternatives do exist. These include online repositories and databases such as the Dataverse Network Project [18]. Specialist data centres or archives are also emerging, such as the UK Data Archive [19]. But in all cases, whether data are published or deposited, restrictions on access to certain aspects of data may be warranted, such as when removal of information that could identify the data would negate its scientific value. In circumstances where data must be behind a barrier to universal access, the data could be made accessible only to those who agree to certain conditions of use, and to individuals who meet certain professional criteria. Embargoes on access to data could also be applied [3].

### Limitations of this guidance

This guidance is directed at quantitative research data and should be applicable to most observational studies and randomised controlled trials. Qualitative or mixed methods researchers should seek alternative advice. The UK Data Archive, for example, has produced guidance on anonymisation techniques for qualitative data [20]. An important limitation of the search strategy used in preparing Table 1 is that it is restricted to US HIPAA guidance and known UK bodies with an interest in maintaining confidentiality in human subjects research. Investigation of requirements of non-English speaking nations would be beneficial. This guidance is aimed at those producing data: guidelines for use of published data have been reported separately [5].

Some advocates of clinical data sharing are also keen for data to be shared in agreed, standardised formats to facilitate its automated re-use for statistical analysis [21]. Although basic principles of data preparation have been provided, how this relates to initiatives aimed at standardising raw data are beyond the scope of this document.

## Competing interests

IH and MN are employees of BioMed Central, the open access publisher, and receive fixed salaries. AJV is an Associate Editor for *Trials*, which hopes to encourage greater prevalence of raw clinical data sharing and publication; DGA is co-Editor-in-Chief of *Trials*. All authors are supporters of data sharing and release from all types of research.

## Authors' contributions

The idea for the manuscript was conceived at a meeting (described by Groves[2] and Hrynaszkiewicz et al[3]) in September 2008 chaired by IH, and was attended by MLN, AJV, and DGA. IH wrote the first draft of the manuscript. MLN, AJV, and DGA all reviewed the manuscript and were involved in its critical revision before submission. All authors read and approved the final manuscript.
